# findWormz is a user-friendly automated fluorescence quantification method for *C. elegans* research.

**DOI:** 10.17912/micropub.biology.001562

**Published:** 2025-04-24

**Authors:** Elizabeth Kitto, John Dean, Scott Leiser

**Affiliations:** 1 University of Michigan–Ann Arbor, Ann Arbor, Michigan, United States; 2 Independent Researcher. deanjohn.pub@gmail.com

## Abstract

The nematode *Caenorhabditis elegans* is a powerful model organism for fluorescent imaging studies due to its simplicity, transparency, well-characterized anatomy, and ease of genetic manipulation. However, the scale and statistical power of *C. elegans* imaging experiments can be limited by the time and effort required to manually quantify fluorescence intensity in individual worms. Recent advances in automated image analysis have used artificial intelligence models and user-supplied training data sets to automate biological image quantification. While these tools have the potential to significantly expedite a variety of research applications in *C. elegans* and other model organisms, they can be difficult to implement and troubleshoot, particularly for researchers with little or no computational training. Here, we introduce a simple method to automate *C. elegans* fluorescence quantification that is accessible to users able to install the free program R and edit a single line of code described here.

**Figure 1. The findWormz method automatically identifies and quantifies the fluorescence intensity of individual C. elegans.**
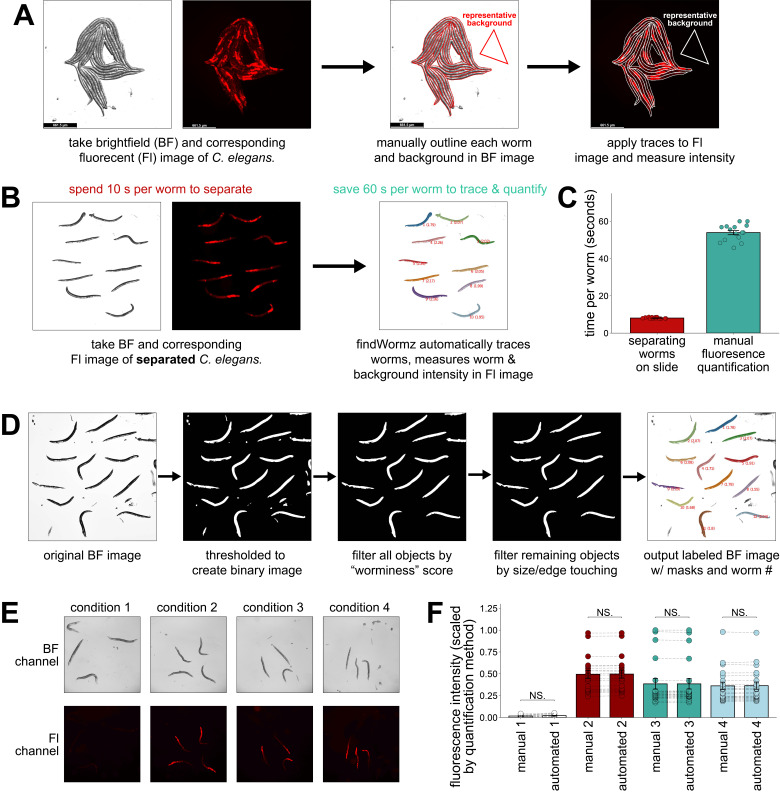
( **A** ) Schematic of a traditional workflow for manually tracing and quantifying the fluorescence of individual *C. elegans* . ( **B** ) Schematic of the findWormz workflow for automated quantification of *C. elegans* fluorescent microscopy images. ( **C** ) Average time in seconds/worm required for a *C. elegans* researcher to separate each worm in a clump of 20 worms and to trace the outline of each worm in an image of 20 clumped worms. ( **D** ) A diagram showing the steps findWormz implements to separate pixels containing worms from the image background. ( **E** ) Representative brightfield (BF) and fluorescent (Fl) channel images of *fmo-2p::mCherry* worms on four conditions with varying fluorescence. ( **F** ) Quantification of images of *fmo-2p::mCherry* worms on four conditions with varying fluorescence. The same images were quantified manually (method described in panel **A** ) or using the findWormz method (described in panel **B** ), and the arbitrary units from the same image analyzed by each quantification method were scaled from 0 to 1. Dashed lines show the same individual worm quantified with each method. Two-way ANOVA with Tukey Post-Hoc pairwise analysis was used to derive p-values. All error bars shown in the figures represent the standard error of the mean (SEM). NS. indicates not significant.

## Description

Fluorescent imaging in *C. elegans* has been widely used to study processes such as neurobiology, development, aging, and stress responses (Corsi et al., 2015; Hutter, 2006). Increasingly sophisticated image analysis techniques have significantly improved the ability to quantify and interpret fluorescent data in *C. elegans* . Open-source software like ImageJ/Fiji (Schneider et al., 2012) and specialized software such as the WormToolbox component of CellProfiler (Wählby et al., 2012) offer tools for segmenting worm shapes and detecting fluorescence in specific regions of interest. Machine learning algorithms and deep learning-based frameworks are also being developed to automate the analysis of complex imaging applications, such as identifying cellular structures or tracking fluorescence changes over time (Bonnard et al., 2022; Jung, 2021; Pan et al., 2024). However, these tools can be difficult to implement and troubleshoot, particularly for researchers with little or no computational training (Carpenter & Singh, 2024; Mangul et al., 2019). An ideal user-friendly automated image analysis tool should 1) run on freely available and robust software, 2) not require training of an artificial intelligence model, 3) require users to edit as little code as possible, and 4) have a small number of adjustable parameters that can be manipulated to suit a wide variety of inputs. We developed the R-based findWormz method to meet these requirements and provide a simple and effective automated fluorescence quantification system.

Many *C. elegans* researchers quantify fluorescent images manually using open-source platforms like ImageJ/FIJI (Bennett et al., 2014; Kumsta et al., 2014; Miller et al., 2022; Schindelin et al., 2012; Wu et al., 2021). This process typically involves tracing the outlines of individual worms in a brightfield image, applying the trace pattern to corresponding fluorescent images, and measuring the mean fluorescence intensity of each worm. A user-selected portion of the image background is also traced and the corresponding region measured in the fluorescent channel to subtract the images' fluorescent background. The resulting data are then manually formatted and transferred into data analysis software for analysis ( **Fig. 1A** ). This method, while functional and simple, requires researchers to manually draw around each individual worm in their sample, limiting maximum potential sample size, statistical power, and experimental scope. By contrast, the findWormz fluorescence quantification method requires slightly more upfront time when taking fluorescent images of *C. elegans* but saves time by automatically identifying and quantifying the fluorescence of each worm in each image of the experiment ( **Fig. 1B** ).

While the transparency of nematodes is beneficial for the ease of imaging fluorescent markers, this transparency does make it difficult for image analysis software to distinguish between multiple worms that are touching. Specifically, their transparency and the backlit background of a standard brightfield microscope create very light and very dark patches within the same worm, depending on what tissues the brightfield light passes through. Consequently, worms do not appear as uniform grey shapes with a dark or light outline, making clumps of touching worms difficult to accurately segment. This challenge can be overcome, but the currently available software that can distinguish between multiple worms touching one another requires training an AI deep learning model for each use case (Wählby et al., 2014; Wählby et al., 2012). Consequently, the findWormz method requires that users take images of worms that have been pushed apart and are not touching in the input image. While this requires more time on the front-end to separate a clump of worms, these extra 10 seconds/worm are generally worth the 60 seconds/worm saved from eliminating the manual worm tracing step ( **Fig. 1C** ). After taking images of separated worms in the brightfield and fluorescent channels, users can place these images in a folder with a key matching file names to experimental conditions.

In order to run findWormz on these images, users will need to download and install the free program R. Detailed instructions and links to downloading R can be found in the full protocol in the extended data. After installing R, the user can open the worm_batch.R file, and edit one line of code to tell the program the location of the images and program files on their computer. This edit involves copying the file path to where their experiment is located on their computer, and pasting this file path into line 13 of the worm_batch.R file to replace the placeholder text labeled "filepath".

After the file path to the *C. elegans* images is specified, the findWormz pipeline begins by opening the brightfield image, enhancing the contrast, correcting for non-uniform background lighting, and blurring the image slightly to increase color uniformity within each worm. Then, findWormz thresholds this corrected brightfield image into a binary image and fills small holes within larger regions of black or white to reduce noise ( **Fig. 1D** , steps 1-2). Next, each object within the binary image is scored for its “worminess”, or the perimeter of the object divided by the 4 times the square root of the object's area. This resulting worminess score is a scale invariant measure reflecting how spindly or round an object is. The lowest possible worminess score, 0.89, would represent a circle. Given their consistently long and thin shape, *C. elegans* have a default worminess score between 1.5-2.1, but the upper and lower bounds of acceptable worminess can be adjusted by the user to account for morphological differences in *C. elegans* mutants. Objects that fall outside of this worminess range are removed from the analysis ( **Fig. 1D** , step 3) **.**

Finally, the remaining objects are filtered by size. Users can adjust the minimum acceptable size range for a worm, depending on the magnification of the image ( **Fig. 1D** , step 4). After this step, the pixel masks of remaining objects are applied to the corresponding fluorescent image and measures of mean fluorescence, standard deviation of fluorescence, worm area, and worm perimeter are recorded. A fluorescent background is also calculated for the image by taking an average intensity measurement of the pixels not identified as worm or debris after initial thresholding (step 2). A colored representation of each identified worm is also plotted onto the original brightfield image, along with a worm number corresponding to each measurement recorded in the analysis spreadsheet ( **Fig. 1D,** step 5). This color-coded image is saved for users to manually run quality control and ensure all recorded objects are, in fact, worms. Details about image quantification and analysis using the findWormz method, as well as the adjustable parameters can be found in the detailed protocol included in the extended data files. All annotated code files can also be found in the extended data files and on Github at https://github.com/eskitto/findWormz.git.

When the findWormz method was tested against the lab “gold-standard” technique of manually tracing worms from the same image, the results were consistent across four different images varying in fluorescence intensity ( **Fig. 1E- F** ). In addition to the above tests for accuracy, the findWormz method has been used successfully in-house for over four years by students and scientists with varying levels of computational training. Together, these results indicate that findWormz is an accurate, user-friendly, and efficient method for quantifying fluorescent images in *C. elegans* . However, the findWormz workflow does still require some technical knowledge. The requirement of installing and editing R code makes findWormz less accessible than completely code-free workflows, such as Image J plug-ins. Nevertheless, running findWormz directly on R allows the program to access 3 GB of working memory rather than the maximum of 1.8 GB in Image J. As a result, the direct use of R prevents the program from running out of memory when analyzing large image sets, and increases analysis speed. Future work in this area could re-write the findWormz analysis process in Java to allow for findWormz to run as an Image J plugin, while keeping in mind that this may restrict analysis capacity.

Together, our results indicate that the findWormz method has the potential to save *C. elegans* researchers about 15 minutes of worm-tracing time per fluorescence image with a standard sample size of 20 worms. Additionally, findWormz measures a more accurate background fluorescence reading than manual methods by including all pixels in the image that are not covered by worms, eggs, larva, or artifacts on the slide. Finally, this method can be run on the free coding platform R and only requires users to modify one line of code under standard imaging conditions. Overall, the findWormz method presents an efficient, user-friendly alternative to manual quantification of *C. elegans* fluorescence images.

## Methods

Strains and growth conditions

Standard *Caenorhabditis elegans* cultivation procedures were used as previously described (Kaeberlein et al., 2006; Smith et al., 2008). Briefly, *fmo-2p::mCherry* fluorescent reporter (LZR01) worms were maintained on solid nematode growth media (NGM) seeded with 200 μL live *E. coli* OP50 (OD _600_ 3.0) throughout life and housed in a 20°C Percival incubator. All experiments were conducted at 20°C unless stated otherwise.

Slide Microscopy

Fluorescent images in this study were taken using Leica Application Suite X (LASX version 3.7.5.24914) software and a Leica scope with >15 worms per treatment at ≥35x magnification. For representative images displayed in the figures, worms were paralyzed in 0.5 M sodium azide (NaN _3_ ) and imaged once residual liquid evaporated and the worms clumped together. For quantification of these images and for imaging screens, the same method was used but after the residual sodium azide had evaporated, individual worms were gently pushed apart until they were no longer touching one another. Fluorescent mean comparisons were then quantified using the findWormz method in R (see extended data for protocol and code) to identify individual worms and measure fluorescent intensity. The mean fluorescent intensity of the background (all non-worm pixels) was subtracted from the mean fluorescence of each worm. All outputs from this R code were visually inspected and any non-worm objects that were misidentified by the program were removed. Data were plotted by R version 4.3.3 and Adobe Illustrator 2022. The brightness of representative fluorescent images in each figure panel was increased to an equal degree across all images within the panel in ImageJ bundled with 64-bit Java 1.8.0.

Statistics and reproducibility

Two-way ANOVA with Tukey Post-Hoc analysis was used to derive p-values for fluorescence comparisons. All error bars shown in the figure represent the standard error of the mean (SEM).

Software Version Information

All R code was tested on R version 4.4.3.tar.gz, and R Studio version 2024.12.1+563.

## Data Availability

Description: detailed protocol for using the findWormz method. Resource Type: Text. DOI: https://doi.org/10.22002/gf5s0-ykx98 Description: R code for using findWormz. Resource Type: Software. DOI: https://doi.org/10.22002/rc50a-dr906
